# Effects of canagliflozin on kidney oxygenation evaluated using blood oxygenation level-dependent MRI in patients with type 2 diabetes

**DOI:** 10.3389/fendo.2024.1451671

**Published:** 2024-08-30

**Authors:** Katsuhito Mori, Tsutomu Inoue, Yuri Machiba, Hideki Uedono, Shinya Nakatani, Masahiro Ishikawa, Satsuki Taniuchi, Yutaka Katayama, Akira Yamamoto, Naoki Kobayashi, Eito Kozawa, Taro Shimono, Yukio Miki, Hirokazu Okada, Masanori Emoto

**Affiliations:** ^1^ Department of Nephrology, Graduate School of Medicine, Osaka Metropolitan University, Osaka, Japan; ^2^ Department of Nephrology, Faculty of Medicine, Saitama Medical University, Saitama, Japan; ^3^ Department of Metabolism, Endocrinology and Molecular Medicine, Graduate School of Medicine, Osaka Metropolitan University, Osaka, Japan; ^4^ School of Clinical Engineering, Faculty of Health and Medical Care, Saitama Medical University, Saitama, Japan; ^5^ Department of Medical Statistics, Graduate School of Medicine, Osaka Metropolitan University, Osaka, Japan; ^6^ Department of Radiology, Osaka Metropolitan University Hospital, Osaka, Japan; ^7^ Department of Diagnostic and Interventional Radiology, Graduate School of Medicine, Osaka Metropolitan University, Osaka, Japan; ^8^ Department of Radiology, Faculty of Medicine, Saitama Medical University, Saitama, Japan

**Keywords:** SGLT2 inhibitor, kidney oxygenation, kidney disease, type 2 diabetes, GFR, BOLD MRI

## Abstract

**Background:**

Recent clinical studies suggest protective effects of SGLT2 inhibitors on kidney disease outcome. Chronic hypoxia has a critical role in kidney disease development, thus we speculated that canagliflozin, an SGLT2 inhibitor, can improve kidney oxygenation.

**Methods:**

A single-arm study was conducted to investigate the effects of canagliflozin on T2* value, which reflects oxygenation level, in patients with type 2 diabetes (T2D) using repeated blood oxygenation level-dependent MRI (BOLD MRI) examinations. Changes in cortical T2* from before (Day 0) to after single-dose treatment (Day 1) and after five consecutive treatments (Day 5) were evaluated using 12-layer concentric objects (TLCO) and region of interest (ROI) methods.

**Results:**

In the full analysis set (n=14 patients), the TLCO method showed no change of T2* with canagliflozin treatment, whereas the ROI method found that cortical T2* was significantly increased on Day 1 but not on Day 5. Sensitivity analysis using TLCO in 13 well-measured patients showed that canagliflozin significantly increased T2* on Day 1 with no change on Day 5, whereas a significant improvement in cortical T2* following canagliflozin treatment was found on both Day 1 and 5 using ROI.

**Conclusions:**

Short-term canagliflozin treatment may improve cortical oxygenation and lead to better kidney outcomes in patients with T2D.

## Introduction

Sodium-glucose cotransporter 2 (SGLT2) inhibitors were originally developed to improve glycemic control in patients with type 2 diabetes (T2D). In previous clinical trials conducted to evaluate cardiovascular safety, SGLT2 inhibitor administration resulted in significant reductions in cardiovascular events (1–3). Furthermore, results of secondary and exploratory analyses of findings obtained in those trials suggested protective effects of SGLT2 inhibitors on kidney outcomes (2–4). More recently, the CREDENCE trial assessed the effects of canagliflozin, an SGLT2 inhibitor, on renal composite outcomes as a primary endpoint, and those results showed kidney-protective effects in patients with T2D and chronic kidney disease (CKD) (5). There is increasing evidence that the kidney-protective effects of SGLT2 inhibitors are independent of glycemic control, though the underlying mechanisms remain largely unknown.

Human kidneys are susceptible to hypoxic damage (6). Once a level of irreversible injury is reached, sustained chronic hypoxia in the tubulointerstitium leads to interstitial fibrosis, resulting in end-stage kidney disease regardless of the original related disease (7). To protect against the so-called ‘final common pathway’, improvement of kidney oxygenation is considered to be a potential useful approach.

Recent technological advancements have enabled non-invasive evaluation of renal oxygenation using blood oxygenation level-dependent magnetic resonance imaging (BOLD MRI) (8, 9), a functional MRI technique. Based on inherent differences in the magnetic properties of deoxy-hemoglobin (Hb) and oxy-Hb, BOLD MRI results can be used to evaluate tissue oxygenation. Increased deoxy-Hb in blood vessels changes the magnetic properties of Hb, resulting in increased R2* (decreased T2*) signals (T2* = 1/R2*), thus higher T2* values (= lower R2*) indicate greater levels of tissue oxygenation. Two recent observational studies independently demonstrated that low cortical oxygenation, i.e., high R2* (10) and low T2* (11), at the baseline indicated CKD progression. In addition, another study examined various multiparametric MRI sequences and found that T2* value was the best predictor for deterioration of estimated glomerular filtration rate (eGFR) (12).

Reabsorption of glucose and sodium in proximal tubular cells requires considerable energy, with a large amount of oxygen consumed (13). Recent mechanistic analysis of results from the CREDENCE trial found that higher glycosuria induced by canagliflozin was strongly linked to lower risk of kidney composite endpoints (14). Therefore, it is speculated that blockade of reabsorption by use of an SGLT2 inhibitor can improve renal tissue oxygenation as a kidney protective mechanism. In this regard, several studies have been conducted to examine the effects of SGLT2 inhibitors on renal oxygenation shown by BOLD MRI (15–19), though the results are inconsistent.

For the present study, the short-term effects of canagliflozin on kidney oxygenation were investigated using results obtained with repeated measurements of BOLD MRI in patients with T2D. The subjects were hospitalized during the trial, which was considered helpful to minimize the influence of potential confounding factors such as salt intake and hydration status (20).

## Methods

### Study design

This was a single-arm, interventional study performed to examine the effects of canagliflozin on kidney oxygenation evaluated using BOLD-MRI in patients with T2D. The protocol was reviewed and approved by the Osaka Metropolitan University Hospital Certified Review Board (approval no. CRB52000004) (Osaka Diabetes Mellitus and Kidney Diseases study-8: Diamond study-8). This trial has been registered in the Japan Registry of Clinical Trials (jRCTs051200047). The study was conducted in accordance with the principles of the Declaration of Helsinki and Ethical Guidelines for Medical and Biological Research Involving Human Subjects by the Ministry of Health, Labor and Welfare of Japan (March 2021). The first subjects were enrolled on September 7, 2020, and the final subject was discharged from the hospital on March 2, 2022.

### Subjects

The subjects were recruited from individuals receiving treatments as an outpatient at Osaka City University Hospital. Age ranged from 20-80 years and each was diagnosed with T2D, with HbA1c of 6.5% or more and less than 10%, without administration of an SGLT2 inhibitor within two weeks (inclusion criteria). Exclusion criteria were 1) pregnancy or breastfeeding, 2) SGLT2 inhibitor drug allergy, 3) contraindications to MRI such as use of pacemaker or claustrophobia, 4) history of frequent urinary and/or genital infections, 4) nephrotic syndrome, 5) use of diuretics and/or non-steroidal anti-inflammatory drugs, 7) severe renal insufficiency (eGFR <30 mL/min/1.73 m^2^), or history of dialysis or kidney transplantation, 8) abnormal renal morphology, such as several cysts, hydronephrosis, or severe calcification, 9) severe liver dysfunction (more than three-fold greater than upper limit of aspartate and alanine aminotransferase according to facility standards), 10) history of malignant tumor, 11) severe cardiac failure, or 12) participation in clinical studies other than this study, or 13) considered inappropriate for study participation by the primary physician. Following explanation of the study objectives, each participant provided written informed consent. At the screening visit after consent, medical history, physical examination, blood and urine test, and renal ultrasonography results were checked to confirm eligibility based on the inclusion and exclusion criteria.

### Procedures

After obtaining consent, all enrolled participants were hospitalized to avoid the potential impact of sodium, calorie intake, and/or body fluid volume on kidney oxygenation (**Supplementary Figure S1**). Food intake in each subject was maintained at a constant level of approximately 25-30 kcal per ideal body weight (kg), with 60% of the calories from carbohydrates and a salt intake of 6-7 g. To adjust hydration status, 350 mL of water was consumed within one hour before each BOLD MRI examination. The initial BOLD MRI was performed in the evening of admission day (Day -2). Baseline MRI data using T2-weighted imaging (T2WI) and diffusion-weighted imaging (DWI) in addition to BOLD MRI were obtained at two days after admission (Day 0). The next day (Day 1), 100 mg of canagliflozin was orally administered from one to three hours before BOLD MRI, as the blood level of canagliflozin has been shown the peak about one hour after a single dose (time of maximum acute pharmacological effects onset) (21). Thereafter, the participants were given canagliflozin each morning. The fourth BOLD MRI procedure was performed after five days of canagliflozin treatment (Day 5), as the blood concentration was considered to be steady-state within four days after beginning administration (21).

### MRI techniques

BOLD MRI was performed using an Ingenia 3.0-T MRI scanner (Philips Medical Systems, Best, the Netherlands), as previously reported (8, 11, 12). Three coronal sections of a kidney were acquired at each MRI examination. The right kidney was selected for evaluation unless there was a problem, such as large cysts or other anatomical abnormalities, so as to avoid possible artifacts due to gastrointestinal gases or fluctuations in organ position (22). The detailed conditions of the multiple gradient echo sequence were as follows: flip angle 50°, echoes 12 times (4.92-31.98 msec), repetition time 172 msec, and slice thickness 5 mm. A T2* map was generated using the average T2* values from three BOLD MRI images, as previously reported (8, 11, 12). T2* values for each BOLD MRI examination were determined using two methods. First, a twelve-layer concentric object (TLCO), or ‘onion peel’, method was used for objective assessment with low bias. This method semi-automatically divides the renal parenchyma into 12 layers of equal thickness with use of MATLAB, version R2020b (The MathWorks, Natick, MA, USA) (10, 12, 23). The mean T2* values for the first to third layers and the eighth to tenth layers represent cortical and medullary oxygenation, respectively. Second, region of interest (ROI), which indicates the average for T2* values in the cortex, was used. T2-weighted images obtained at the baseline were used for morphologic evaluation as a reference to avoid areas with cysts, mass lesions, or stones at the same level. The ROI was manually defined in the cortex or subcapsular parenchyma when it was difficult to determine the border between the cortex and medulla of the largest cross section of the kidney in advanced CKD cases (11).

DWI was also performed to assess apparent diffusion coefficient (ADC) values for evaluation of kidney fibrosis on Day 0. ADC values were determined using OsiriX MD, version 12.0.3 (Pixmeo, Geneva, Switzerland).

### Study endpoints and assessments

The prespecified primary endpoints were change in T2* value between before and after canagliflozin treatments. T2* values after a single dose (Day 1, about two hours after initial dose) and after five consecutive treatments (Day 5) were compared with those obtained at the baseline (Day 0) using the TLCO (main analysis) and ROI (sub-analysis) methods. Secondary endpoints included changes in erythropoietin, HbA1c, glycated albumin, fasting plasma glucose, urinary albuminuria, urinary proteinuria, and eGFR, as well as others. Safety evaluations included adverse events and clinical laboratory test results.

### Statistical analysis

Statistical analyses of the full analysis set (FAS) were performed without imputation for missing data. Sample size was determined based on a prior study protocol (24). A sample size of 15 participants was considered to provide a power of 90% with a two-sided significance level of 5%, based on oxygenation improved by 10% with treatment. It was anticipated that some participants would discontinue the study, thus the original plan was to enroll 20.

Baseline characteristics were summarized using median values and interquartile range for continuous variables, and percentages for categorical variables. The geometric mean of T2* for each time point was estimated using a generalized least squares based on the assumption that the variance-covariance matrix was compound symmetry. The model included indicator variables for the time points (Day 0, 1, 5) as explanatory variables and log-transformed T2* as the outcome variable. P-value for the global test was calculated based on a null hypothesis, which assumed that the coefficient equaled 0 for all time indicator variables. For sensitivity analysis, subgroup analysis was conducted, with the dataset divided by the eGFR or ADC value. For the outcome of other continuous variables, a Wilcoxon signed-rank test was performed to compare values between each time point.

All statistical analyses were performed with a two-sided significance level of 5% using the R software package, version 4.3.1.

## Results

A flow diagram for the present study is shown in **Supplementary Figure S2**. Initially, the plan was to enroll 20 patients in the study, though there was a delay due to a coronavirus outbreak. In addition, the study used for reference (24) found no significant change of R2* in individuals treated with empagliflozin (15). Therefore, it was decided to finish the present study within the scheduled deadline (March 2022). After screening 16 patients, two were excluded because of liver dysfunction or infection. Finally, 14 were enrolled and completed the planned examinations. The FAS includes findings for all participants. However, it was difficult to determine the T2* value using TLCO in one because of severe kidney atrophy, thus that case was removed for sensitivity analysis. The **Table 1** shows clinical characteristics of the 14 participants, with median age 65.5 (interquartile range 59.0-72.0) years, body mass index 24.2 (22.3-26.7) kg/m^2^, HbA1c 7.1% (6.9-7.7%), eGFR 59.2 (46.9-76.8) mL/min/1.73 m^2^, and urinary albumin 20.5 (5.7-468.7) mg/day. Representative T2-weight, T2* map, and quantitative evaluation images are shown in **Supplementary Figure S3**.

**Table 1:** Clinical characteristics of subjects.

Age, years	65.5 (59.0-72.0)
Male/female (%)	10/4 (71/29)
Body mass index, kg/m^2^	24.2 (22.3-26.7)
Systolic blood pressure, mmHg	126.0 (109.5-131.2)
Hemoglobin, g/dL	13.9 (13.1-15.2)
Albumin, g/dL	3.9 (3.9-4.2)
Blood urea nitrogen, mg/dL	14.5 (13.2-17.8)
Creatinine, mg/dL	0.93 (0.74-1.28)
Estimated GFR, mL/min/m^2^	59.2 (46.9-76.8)
Fasting plasma glucose (FPG), mg/dL	119.5 (105.2-158.5)
HbA1c (%)	7.1 (6.9-7.7)
Glycated albumin (%)	18.4 (16.6-19.6)
Urinary albumin, mg/day	20.5 (5.7-468.7)
Medications	
DPP-4 inhibitor (%)	8 (57)
Metformin (%)	6 (43)
GLP-1 receptor agonist (%)	1 (7)
Glinide (%)	1 (7)
Insulin (%)	1 (7)
ACE inhibitor or ARB (%)	7 (50)
Statin (%)	11 (79)

Data are shown as the median (interquartile range) or number (%).

**Figure 1:**
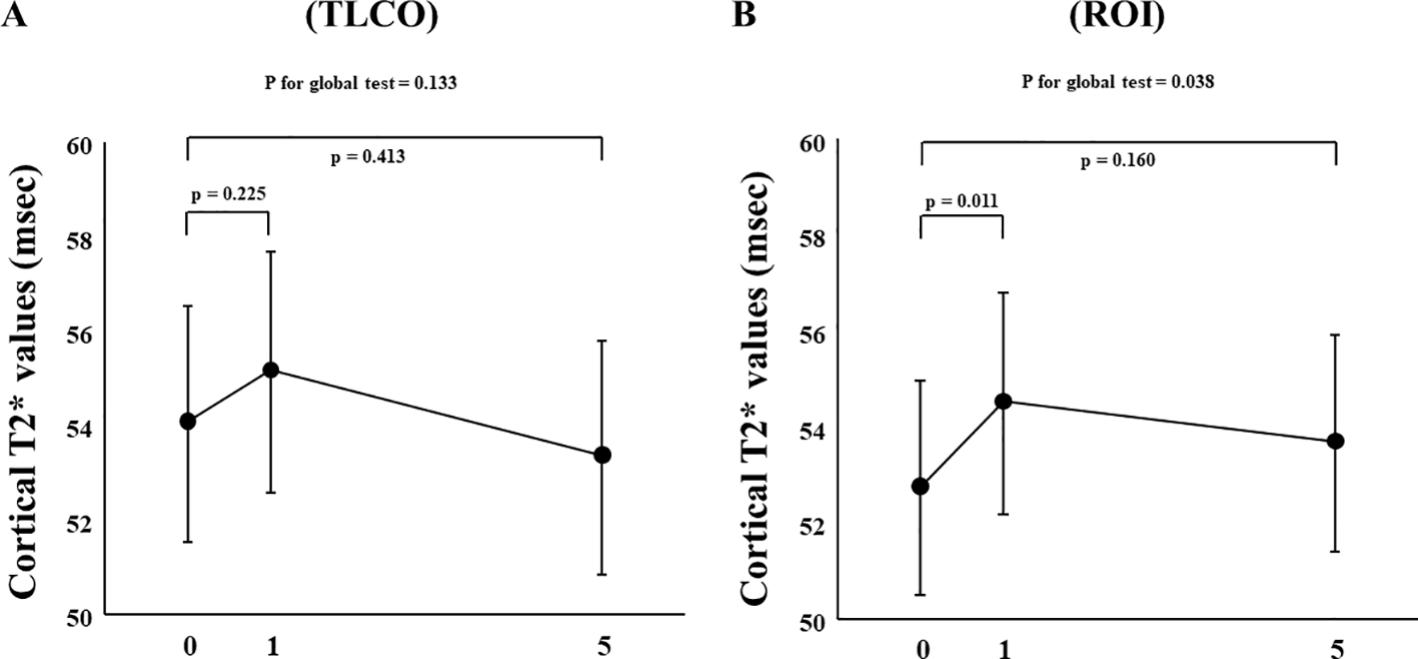
Changes in cortical T2* values from Day 0 (baseline) to Day 1 (initial single-dose canagliflozin treatment) and Day 5 (after five consecutive days of canagliflozin treatment) in FAS using **(A)** TLCO and **(B)** ROI methods. FAS, full analysis set; TLCO, twelve-layer concentric object; ROI, region of interest.

**Figure 2:**
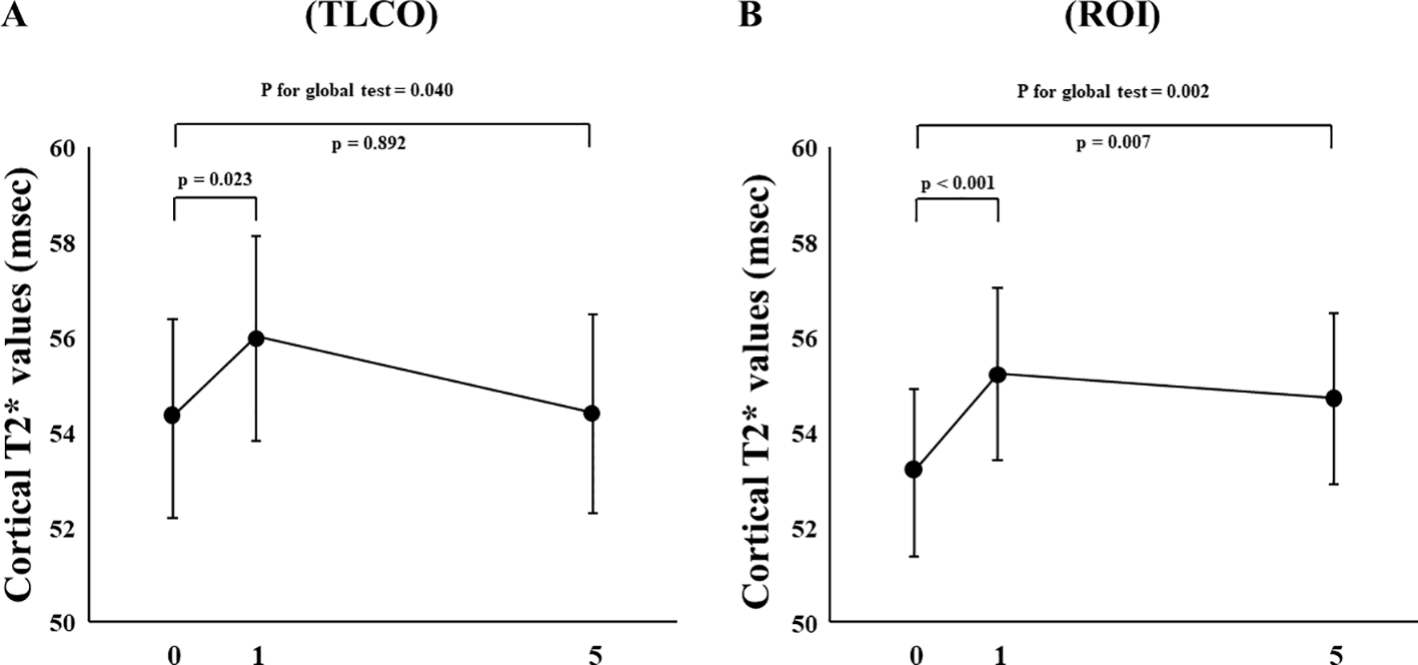
Changes in cortical T2* values from Day 0 (baseline) to Day 1 (initial single-dose canagliflozin treatment) and Day 5 (after five consecutive days of canagliflozin treatment) in sensitivity analysis using **(A)** TLCO and **(B)** ROI methods. TLCO, twelve-layer concentric object; ROI, region of interest.

**Figure 3:**
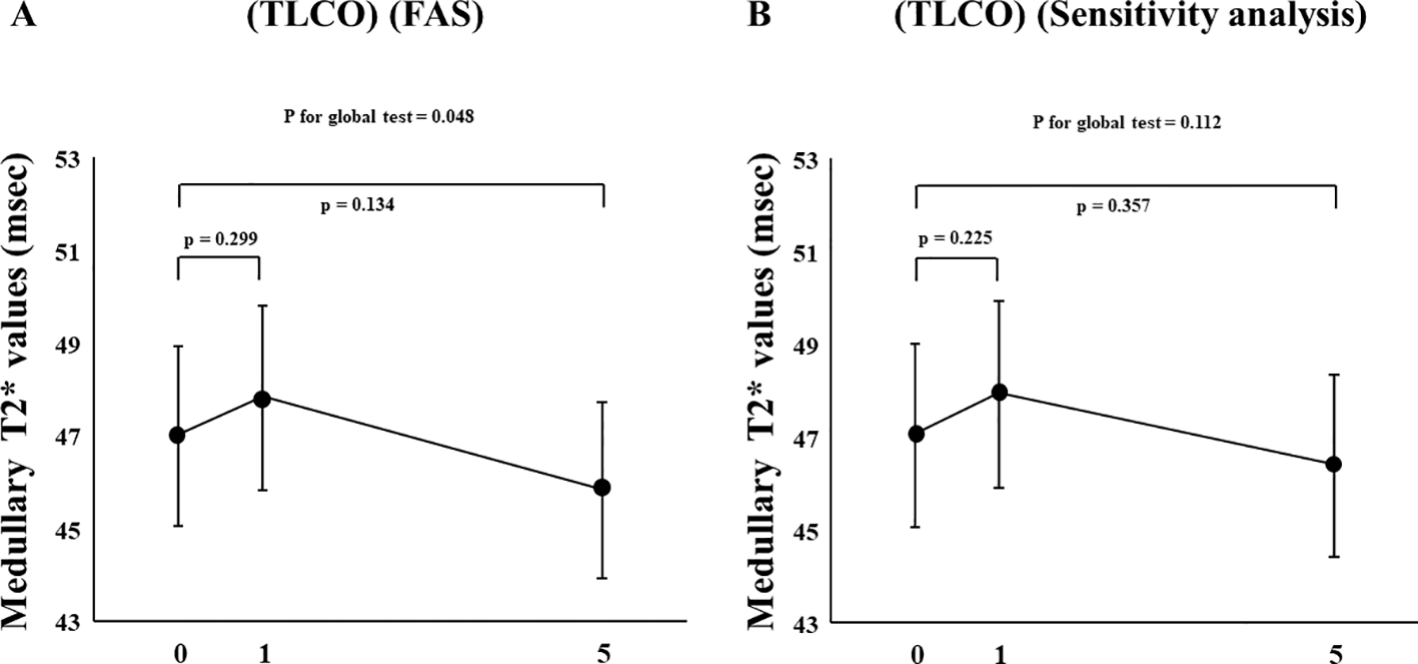
Changes in medullary T2* values from Day 0 (baseline) to Day 1 (initial single-dose canagliflozin treatment) and Day 5 (after five consecutive days of canagliflozin treatment) shown by **(A)** FAS and **(B)** sensitivity analysis using TLCO. FAS, full analysis set; TLCO, twelve-layer concentric object; ROI, region of interest.

In FAS analysis, findings obtained with the TLCO method showed that canagliflozin treatment did not cause a significant change in T2* value from Day 0 to Day 1 or to Day 5 (**Figure 1A**) (**Supplementary Table S1**). On the other hand, with the ROI method, the cortical T2* value was significantly increased from 52.8 (50.6-55.0) to 54.5 (52.3-56.9) (p = 0.011) on Day 1, while no significant change was found from the baseline to Day 5 (**Figure 1B**) (**Supplementary Table S1**). There was no change in T2* from Day -2 to Day 0 before canagliflozin treatment (**Supplementary Figure S4**). Next, sensitivity analysis was performed based on findings for the 13 well-measured participants. Using TLCO, canagliflozin treatment was shown to significantly increase the T2* value from 54.3 (52.3-56.4) to 56.0 (53.9-58.2) (P = 0.023) on Day 1, with no significant change on Day 5 (**Figure 2A**) (**Supplementary Table S1**). Furthermore, ROI method findings indicated a significant improvement in cortical T2* following treatment with canagliflozin from 53.2 (51.4-55.0) to 55.2 (53.5-57.1) (p <0.001) on Day 1 and to 54.7 (53.0-56.6) (P = 0.007) on Day 5 (**Figure 2B**) (**Supplementary Table S1**). As for medullary oxygenation associated with canagliflozin treatment, no significant change in T2* was shown using TLCO in either FAS (**Figure 3A**) (**Supplementary Table S1**) or sensitivity analysis results (**Figure 3B**) (**Supplementary Table S1**).

**Figure 4:**
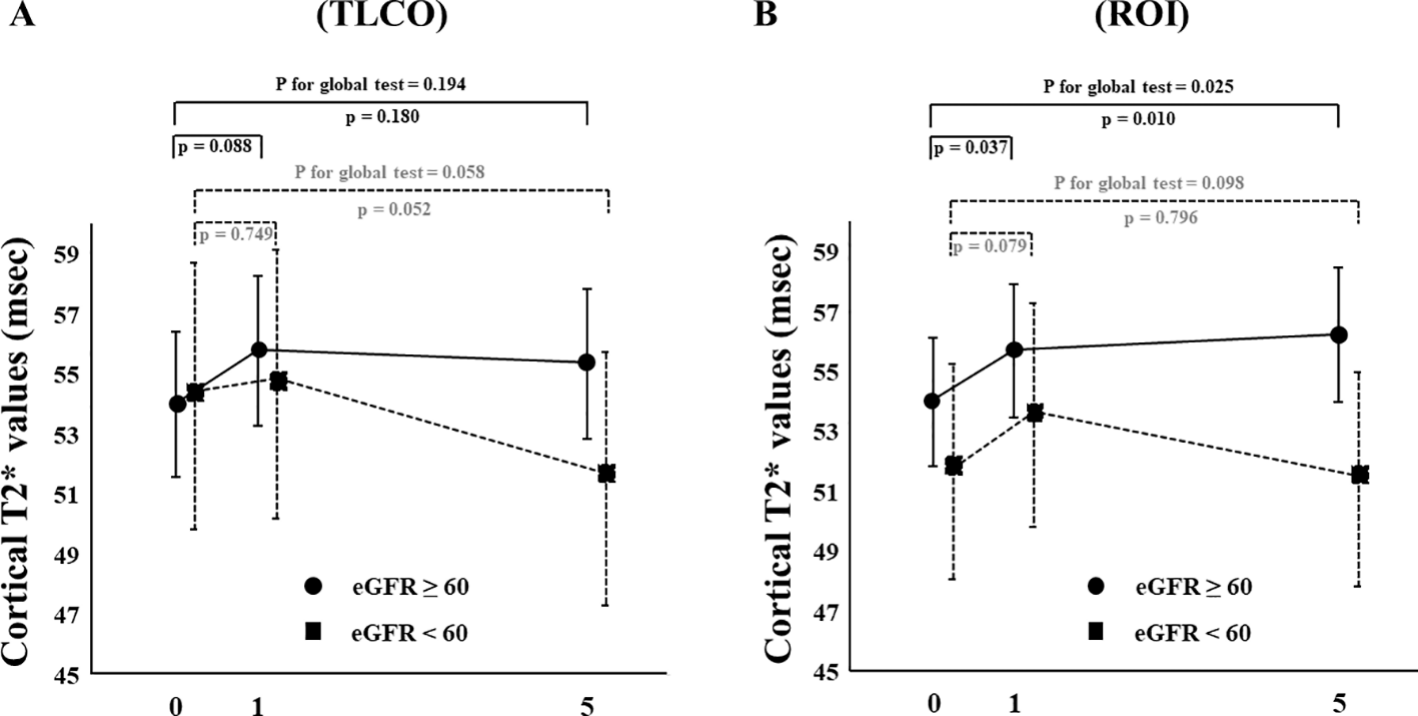
Changes in cortical T2* values from Day 0 (baseline) to Day 1 (initial single-dose canagliflozin treatment) and Day 5 (after five consecutive days of canagliflozin treatment) in FAS using **(A)** TLCO and **(B)** cortical ROI methods, stratified by eGFR ≥60 or <60. FAS, full analysis set; TLCO, twelve-layer concentric object; ROI, region of interest; eGFR, estimated glomerular filtration rate.

**Figure 5:**
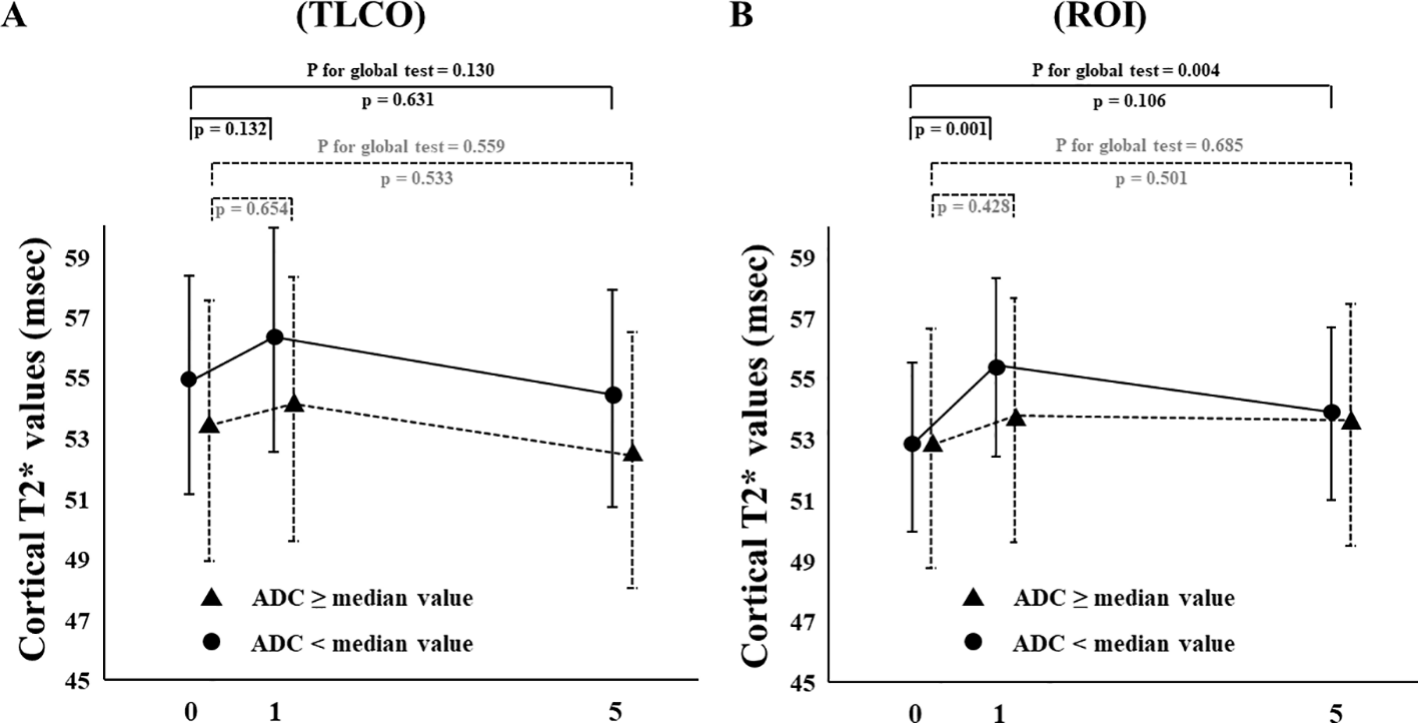
Changes in cortical T2* values from Day 0 (baseline) to Day 1 (initial single-dose canagliflozin treatment) and Day 5 (after five consecutive days of canagliflozin treatment) in FAS using **(A)** TLCO and **(B)** cortical ROI methods, stratified by ≥ or < the median ADC value. FAS, full analysis set; TLCO, twelve-layer concentric object; ROI, region of interest, ADC, apparent diffusion coefficient.

The effects of canagliflozin on cortical T2* value was examined with stratification based on eGFR (low and high groups: eGFR <60 and ≥ 60 mL/min/m^2^, respectively) and ADC (< or ≥ median X10^-3^mm^2^/s) using TLCO. Canagliflozin treatment did not cause a change in T2* values in FAS analysis using the TLCO method in either the low or high eGFR group (**Figure 4A**) (**Supplementary Table S1**). On the other hand, findings obtained with the ROI method showed that canagliflozin significantly increased cortical T2* on both Day 1 and 5 in the high eGFR group (**Figure 4B**) (**Supplementary Table S1**). As for ADC stratification, there was no significant change in T2* caused by treatment with canagliflozin shown by FAS analysis using the TLCO method (**Figure 5A**) (**Supplementary Table S1**). However, with use of ROI, cortical T2* was found to be significantly increased on Day 1 in the low ADC group, which was thought to be related to lower levels of kidney fibrosis (**Figure 5B**) (**Supplementary Table S1**).

As expected, canagliflozin treatment caused an acute decrease in eGFR, known as the initial dip (**Supplementary Table S2**). Furthermore, five-day treatment with canagliflozin significantly improved fasting plasma glucose, glycated albumin, urinary albumin, and urinary protein levels, whereas there was no significant change in erythropoietin level with short-term canagliflozin treatment (**Supplementary Table S2**).

No serious adverse events considered to be related to this clinical trial were observed during the study period.

## Discussion

This is the first study to investigate the effects of canagliflozin on kidney oxygenation in patients under a hospitalized condition throughout an extended period. Following stabilization of hydration status, the short-term effects of such therapy on T2* values were investigated in 14 participants using multiple BOLD MRI examinations. Results obtained with the TLCO method showed that cortical T2* values were not significantly changed with canagliflozin treatment. On the other hand, results with ROI showed that a single dose of canagliflozin significantly improved cortical T2*. Moreover, sensitivity analysis results with use of the TLCO method indicated that the cortical T2* value was significantly increased on Day 1 of canagliflozin use, while ROI results showed significant increases in cortical T2* values on both Day 1 and 5. On the other hand, no significant changes in medullary T2* values caused by canagliflozin were noted. These findings suggest that canagliflozin treatment may improve cortical oxygenation in T2D patients.

Zanchi et al. examined the effects of empagliflozin on kidney oxygenation using BOLD-MRI with TLCO in healthy participants (15). Neither a single dose nor four-week intervention of empagliflozin altered cortical or medullary R2* values. On the other hand, Laursen et al. reported that high-dose dapagliflozin (50 mg) significantly decreased R2*, indicating improved oxygenation, determined by ROI at six hours after administration in patients with type 1 diabetes (T1D) (16). Furthermore, a randomized controlled trial conducted by Zhou et al. showed that a 24-week administration of canagliflozin, as compared with glimepiride, decreased both cortical and medullary R2*, with improved cortical and medullary oxygenation, using the ROI method in newly-diagnosed T2D patients (17), while Gullaksen et al. reported that treatment with empagliflozin for 32 weeks in T2D patients significantly reduced medullary oxygenation but not cortical oxygenation by TLCO (18). In contrast, in a recent study reported by Zhang et al., six-week empagliflozin treatment increased medullary oxygenation without cortical change in T2D patients (19). These inconsistent findings may be related to variations among the subjects, intervention periods, and measurement methods (ROI or TLCO) employed in those studies. Additionally, such discrepancies among related studies might have originated from difficulties with adjustment of hydration status, due to varying amounts of salt, water, and calorie intake. For the present investigation, the short-term effects of canagliflozin on kidney oxygenation were carefully evaluated by repeated BOLD MRI examinations with both TLCO and ROI methods in T2D patients under a hospitalized condition. It was found that a single dose of canagliflozin administered to a patient with T2D, considered to reflect pharmacological effects, may increase cortical oxygenation through direct blockade of SGLT2. This finding is compatible with previously reported results showing that single-dose dapagliflozin-induced improvement of cortical oxygenation in T1D patients (16).

Glucose reabsorption in the proximal tubules is an energy- and oxygen- consuming process (13). The Na/K ATPase enzyme is the driving force of SGLT2. Na/K ATPase on the basolateral side functions to pump sodium out of proximal tubular cells and, consequently, provides for inward sodium movement, which drives intracellular glucose co-transport through SGLT2 on the luminal side. Intracellularly accumulated glucose is transported into blood vessels via GLUT2 on the basolateral side in a concentration-dependent manner. Since ATP synthesis is associated with oxygen consumption, it is speculated that SGLT2 inhibitors decrease ATP requirement and oxygen consumption. A previous study showed that both SGLT2 and GLUT2 expressions were higher in proximal tubular cells isolated from patients with T2D as compared to those from healthy subjects, suggesting increased glucose reabsorption under a diabetic condition (25). Also, an experimental study used insertion of an electrode into the kidney parenchyma of streptozotocin-induced diabetic rats to examine whether blockade of glucose reabsorption by phlorizin, which inhibits both SGLT2 and SGLT1, directly affects cortical and medullary oxygenation (26). As expected, those diabetic rats showed decreased cortical oxygenation as compared to non-diabetic control rats, possibly through increased glucose and sodium reabsorption. The authors noted that acute treatment with phlorizin reversed cortical hypoxia in the diabetic rats, whereas SGLT inhibition induced medullary hypoxia in both the diabetic and non-diabetic rats by increased sodium load in more distal segments (26). Those experimental results support other findings showing that single-dose treatment with an SGLT2 inhibitor increases cortical oxygenation in T1D (16) and T2D (this study) patients, but not in healthy subjects (15).

Several mechanisms related to kidney protection by SGLT2 inhibitors have been speculated (13). For example, significant increases in Hb and hematocrit (Ht) have been observed in clinical trials. Mediation analysis findings indicated that increased Hb and Ht inversely predicted cardiovascular events, and those changes seemed to be attributable to an increase in erythropoietin (EPO) level in response to SGLT2 inhibition (27). As described above, Gullaksen et al. reported that long-term treatment with empagliflozin for 32 weeks significantly reduced medullary but not cortical oxygenation (18). They suggested that chronic SGLT2 inhibition could induce medullary hypoxia and subsequent induction of EPO, resulting in kidney protection. However, such a putative mechanism was not likely induced in the present subjects, as no significant change in EPO level after five-day treatment with canagliflozin was noted. Another possible mechanism may be improvement of hyperfiltration through tubuloglomerular feedback (13). SGLT2 inhibitors are known to transiently decrease eGFR, with the initial dip considered to reflect reduction in intraglomerular pressure. Indeed, such an initial dip was noted in the present study. In contrast to increases in T2* noted on Day 1, T2* on Day 5 was increased to a lesser extent. Laursen et al. reported that cortical oxygenation was improved by a single-dose administration of dapagliflozin without any change in blood flow or perfusion in the kidneys (16). Therefore, such hemodynamic change may have veiled the increase in T2* after five days of canagliflozin treatment, as compared to the direct pharmacological effects noted on Day 1.

A deeper examination of analysis results stratified by eGFR and ADC might provide insight into the effects of canagliflozin on cortical oxygenation. The present findings showed that canagliflozin tended to increase cortical T2* in patients with greater preserved kidney function (**Figure 4**) and lower amount of fibrotic change (**Figure 5**). A limited response to canagliflozin in patients with lower eGFR and higher fibrosis could originate from either advanced kidney disease or the detection limit of BOLD MRI. Future studies will be necessary to differentiate between these possibilities.

The present study has both strengths and limitations. The effects of canagliflozin on kidney oxygenation were carefully assessed by repeated BOLD MRI examinations using both TLCO and ROI methods. Standardization of baseline hydration status is known to be critical for objective evaluation using BOLD MRI (20). This study is the first to analyze measurements of kidney oxygenation under hospitalized conditions throughout an extended period. This approach allowed for adjustments of water, salt, and calorie intake, resulting in a more accurate evaluation of kidney oxygenation. Furthermore, the enrolled T2D patients had broad ranges of age, kidney function, and albuminuria. As compared to previous studies, the subjects had higher age and lower eGFR without overt proteinuria, which might reflect patients seen in real-world clinical practice. Although TLCO is an objective method, unexpectedly, it was not possible to separate the kidney parenchyma into 12 layers in a few cases because of severe kidney atrophy. A prespecified statistical method for this precise clinical trial was data analysis using FAS without any imputation. One subject with a severely atrophic kidney showed renal parenchymal thickness much less than 12 pixels in length, making it difficult to measure with the TLCO method, and it was considered that those results should be excluded from analysis, in consideration of the purpose of this study and its exploratory design. Thus, sensitivity analysis may provide more accurate results regarding the effects of canagliflozin on kidney oxygenation. A major limitation is the low number of subjects. In addition, the single arm design without a control group may be another limitation, though repeated BOLD MRI examinations were performed. In addition, direct GFR, blood flow, and renal perfusion were not measured.

In conclusion, short-term treatment with canagliflozin may improve cortical oxygenation, as assessed by BOLD MRI, in patients with T2D. An increase in oxygenation induced by SGLT2 inhibitor administration may have important long-term effects for favorable kidney outcomes.

## Data Availability

The data that support the findings of this study are available from the corresponding author (K.M.) upon reasonable request.
