# Supplementary material for: Effects of canagliflozin on kidney oxygenation evaluated using blood oxygenation level-dependent MRI in patients with type 2 diabetes
Source: Front Endocrinol (Lausanne). 2024 Aug 30;15:1451671. doi: 10.3389/fendo.2024.1451671 (PMC11393780; doi:10.3389/fendo.2024.1451671)

Table S1

Changes in T2* values before and after canagliflozin treatment.

	Day 0	Day 1	Day 5
TLCO cortical T2*<br>(FAS)	54.1 (51.7-56.7)<br>(P for global test=0.133)	55.2 (52.7-57.8)<br>p = 0.225 vs. Day 0	53.4 (51.0-55.9)<br>p = 0.413 vs. Day 0
ROI cortical T2*<br>(FAS)	52.8 (50.6-55.0)<br>(P for global test=0.038)	54.5 (52.3-56.9)<br>p = 0.011 vs. Day 0	53.7 (51.5-56.0)<br>P = 0.160 vs. Day 0
TLCO cortical T2*<br>(Sensitivity analysis)	54.3 (52.3-56.4)<br>(P for global test=0.040)	56.0 (53.9-58.2)<br>p = 0.023 vs. Day 0	54.4 (52.4-56.5)<br>p = 0.892 vs. Day 0
ROI cortical T2*<br>(Sensitivity analysis)	53.2 (51.4-55.0)<br>(P for global test=0.002)	55.2 (53.5-57.1)<br>p <0.001 vs. Day 0	54.7 (53.0-56.6)<br>p = 0.007 vs. Day 0
TLCO medullary T2*<br>(FAS)	47.0 (45.1-49.0)<br>(P for global test=0.048)	47.8 (45.9-49.9)<br>p = 0.299 vs. Day 0	45.8 (44.0-47.8)<br>p = 0.134 vs. Day 0
TLCO medullary T2*<br>(Sensitivity analysis)	47.1 (45.1-49.1)<br>(P for global test=0.112)	48.0 (46.0-50.0)<br>p = 0.225 vs. Day 0	46.4 (44.5-48.4)<br>p = 0.357 vs. Day 0
TLCO cortical T2*<br>(FAS) (eGFR ≥ 60)	53.9 (51.6-56.3)<br>(P for global test=0.194)	55.7 (53.3-58.1)<br>p = 0.088 vs. Day 0	55.2 (52.9-57.7)<br>p = 0.180 vs. Day 0
TLCO cortical T2*<br>(FAS) (eGFR < 60)	54.3 (49.5-59.6)<br>(P for global test=0.058)	54.7 (49.9-60.0)<br>p = 0.749 vs. Day 0	51.6 (47.0-56.6)<br>p = 0.052 vs. Day 0
ROI cortical T2*<br>(FAS) (eGFR ≥ 60)	53.9 (52.0-55.8)<br>(P for global test=0.025)	55.6 (53.6-57.6)<br>p = 0.037 vs. Day 0	56.1 (54.1-58.1)<br>p = 0.010 vs. Day 0
ROI cortical T2*<br>(FAS) (eGFR < 60)	51.7 (47.7-56.0)<br>(P for global test=0.098)	53.5 (49.4-58.0)<br>p = 0.079 vs. Day 0	51.4 (47.4-55.7)<br>p = 0.796 vs. Day 0
TLCO cortical T2*<br>(FAS) (ADC ≥ median)	53.4 (49.2-57.9)<br>(P for global test=0.559)	54.1 (49.9-58.7)<br>p = 0.654 vs. Day 0	52.4 (48.3-56.8)<br>p = 0.533 vs. Day 0
TLCO cortical T2*<br>(FAS) (ADC < median)	54.8 (51.6-58.3)<br>(P for global test=0.130)	56.3 (53.0-59.9)<br>p = 0.132 vs. Day 0	54.4 (51.2-57.8)<br>p = 0.631 vs. Day 0
ROI cortical T2*<br>(FAS) (ADC ≥ median)	52.8 (48.6-57.3)<br>(P for global test=0.685)	53.7 (49.5-58.3)<br>p = 0.428 vs. Day 0	53.6 (49.4-58.1)<br>p = 0.501 vs. Day 0
ROI cortical T2*<br>(FAS) (ADC < median)	52.8 (50.2-55.5)<br>(P for global test=0.004)	55.4 (52.6-58.3)<br>p = 0.001 vs. Day 0	53.8 (51.2-56.6)<br>p = 0.106 vs. Day 0

Data are shown as the geometric mean (95% confidential interval). FAS, full analysis set; TLCO, twelve-layer concentric object; ROI, region of interest; ADC, apparent diffusion coefficient.

Table S2

Changes in various clinical parameters before and after canagliflozin treatment

	Day 0	Day 2	Day 5
eGFR (mL/min/m^2)	59.2 (46.9-76.8)	53.5 (40.5-65.1)<br>p = 0.002 vs. Day 0	53.3 (41.8-65.7)<br>p = 0.001 vs. Day 0
Erythropoietin (IU/L)	7.85 (6.22-9.92)		7.75 (6.25-10.73)<br>p = 1.000 vs. Day 0
Fasting plasma glucose (FPG) (mg/dL)	120 (105-159)	108 (98-128)<br>p = 0.003 vs. Day 0	108 (95-125)<br>p = 0.003 vs. Day 0
HbA1c (%)	7.05 (6.90-7.70)		7.00 (6.80-7.78)<br>p = 0.177 vs. Day 0
Glycated albumin (GA) (%)	18.4 (16.6-19.6)		18.1 (16.1-19.1)<br>p = 0.001 vs. Day 0
Urinary albumin (mg/day)	20.5 (5.7-468.7)	15.4 (6.7-357.9)<br>p = 0.052 vs. Day 0	20.1 (6.8-328.5)<br>p = 0.007 vs. Day 0
Urinary protein (mg/day)	128.0 (73.0-650.0)	358.0 (52.5-469.0)<br>p = 0.029 vs. Day 0	94.0 (55.0-427.0)<br>p = 0.036 vs. Day 0
Urinary sodium (mEq/day)	91.5 (71.2-101.8)	78.0 (70.5-93.2)<br>p = 0.345 vs. Day 0	72.5 (62.0-89.2)<br>p = 0.100 vs. Day 0
Urinary potassium (mEq/day)	31.5 (26.2-34.5)	29.0 (26.0-33.8)<br>p = 0.450 vs. Day 0	30.5 (26.2-36.5)<br>p = 0.826 vs. Day 0
Urinary chloride (mEq/day)	89.0 (78.2-100.2)	81.5 (72.2-94.8)<br>p = 0.615 vs. Day 0	71.0 (62.2-83.2)<br>p = 0.048 vs. Day 0
Urinary β2 microglobulin	93.0 (58.8-156.2)	63.0 (56.0-102.8)<br>p = 0.307 vs. Day 0	112.5 (56.0-151.2)<br>p = 0.476 vs. Day 0
Urinary NAG	2.35 (1.65-3.70)	2.60 (1.63-3.45)<br>p = 0.402 vs. Day 0	2.45 (2.02-3.20)<br>p = 0.328 vs. Day 0

Data are shown as the median (interquartile). P values were obtained using a Wilcoxon signed-rank test.

Figure S1

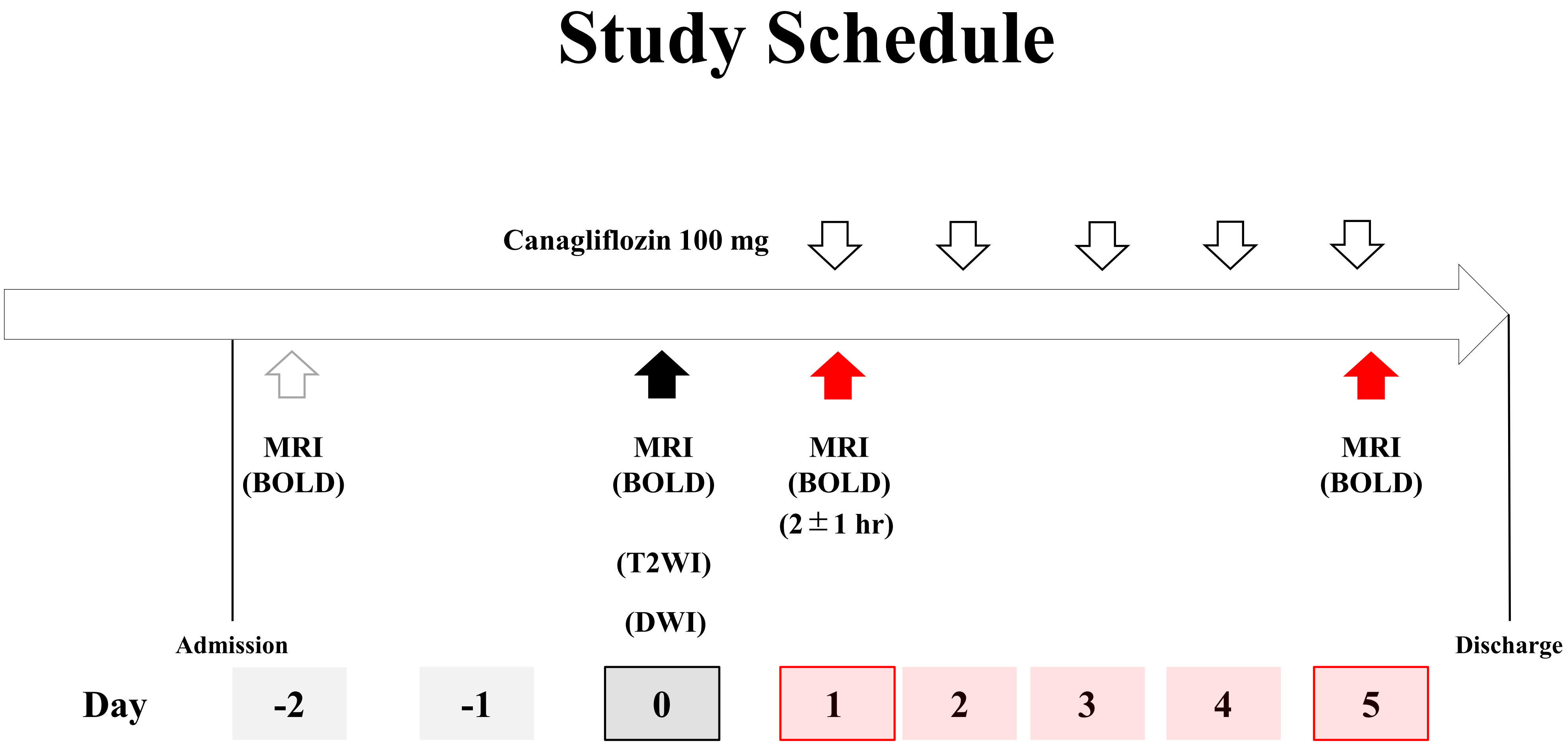

T2WI, T2-weighted image
DWI, diffusion weighted image

Figure S2

Study flow diagram

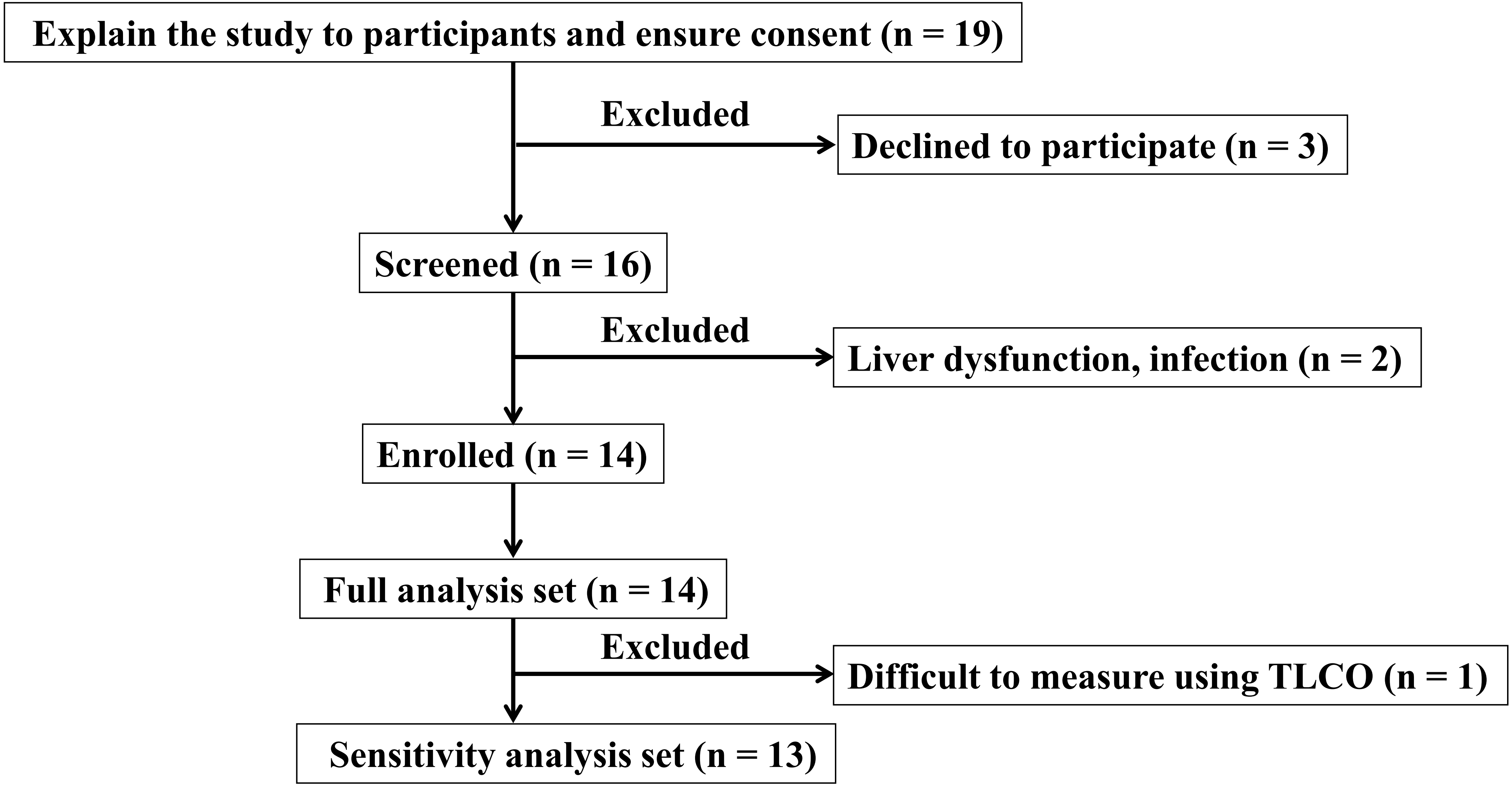

Figure S3

Representative image, T2* map, and quantitative evaluation

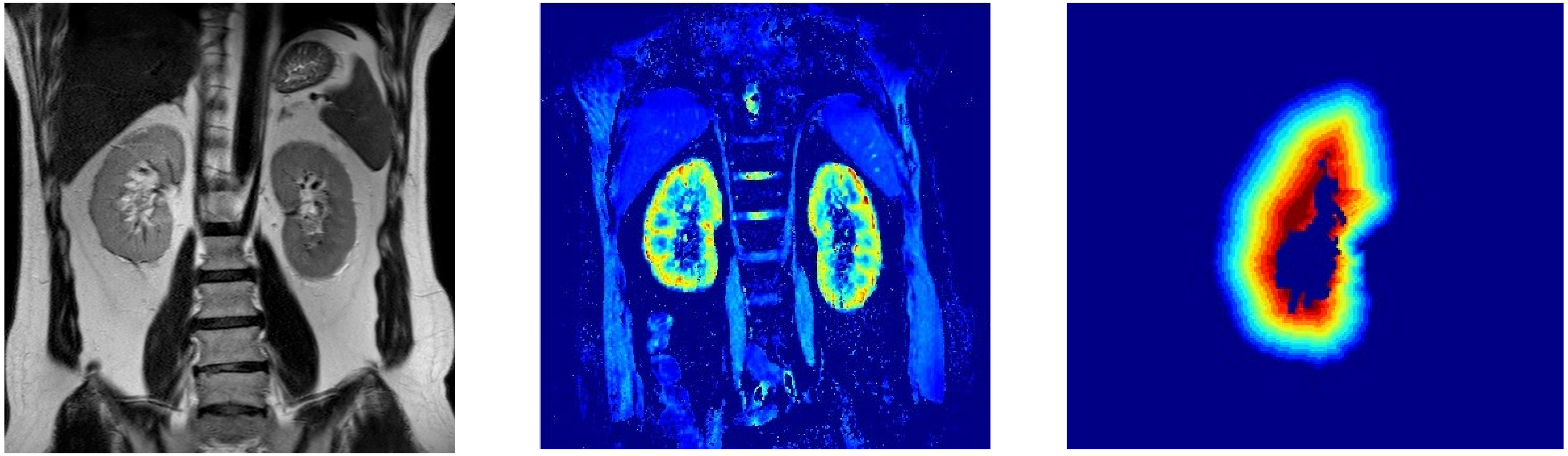

T2-weighted image   T2* map   TLCO

Figure S4

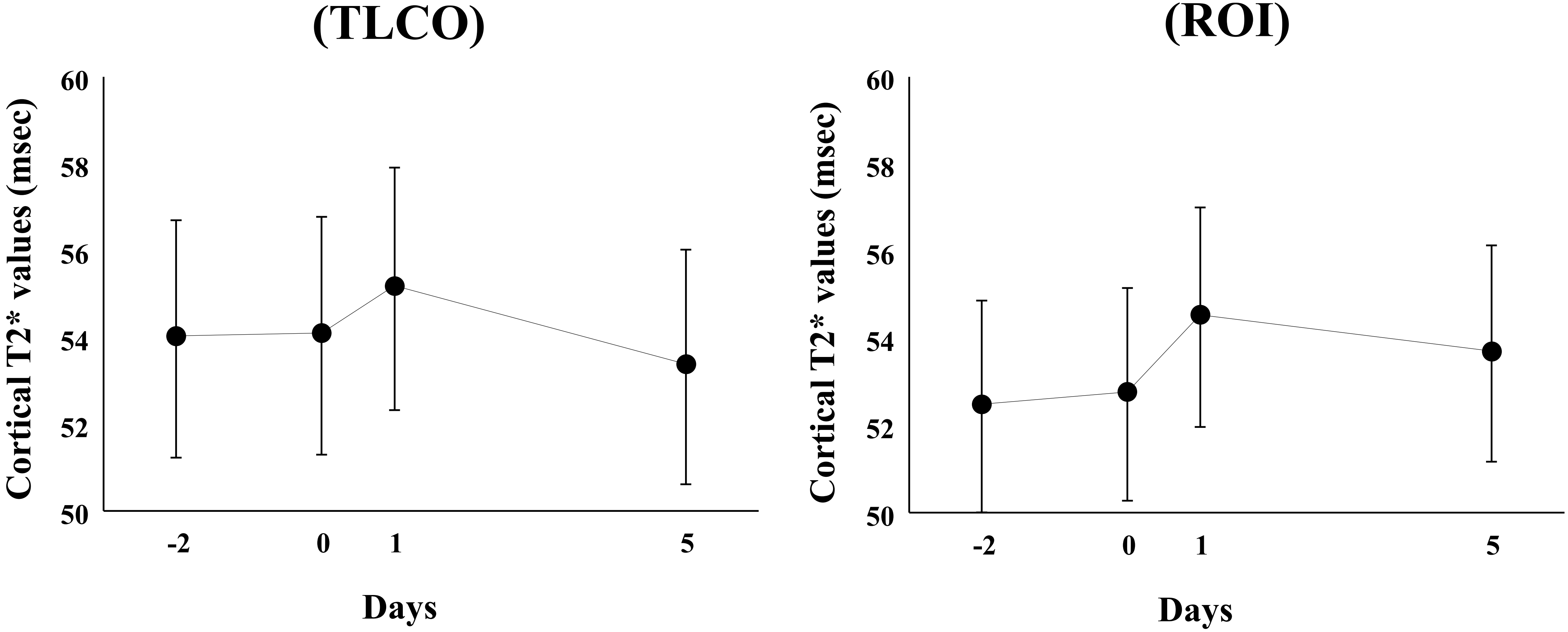

Supplement: Supplementary file 1 [file DataSheet1.pdf]
